# Difference of onset rhythm in acute ischemic stroke patients with different TOAST subtypes

**DOI:** 10.1371/journal.pone.0357414

**Published:** 2026-09-08

**Authors:** Weiqian Li, Lili Zang, Yingyue Zhang, Yuan Gao, Na Ni, Huan Liu, Yanyan Ling, Ming Cheng

**Affiliations:** 1 Department of Basic Medical Sciences, The 960th Hospital of PLA, Jinan, China; 2 Department of Neurology, The 960th Hospital of PLA, Jinan, China; 3 Department of Neurology, Shandong Second Provincial General Hospital, Jinan, China; Essen University Medical School, GERMANY

## Abstract

**Objective:**

To delineate the onset changes of acute ischemic stroke (AIS) patients with different Trial of Org 10172 in Acute Stroke Treatment (TOAST) subtypes in circadian rhythm, seasonal distribution, and weekdays/holidays.

**Materials and methods:**

Patients with AIS admitted to the Emergency Stroke Center of the 960th Hospital of PLA between 1/4/2019 and 30/12/2024 were enrolled as study subjects. Based on medical history and examination results, enrolled patients were classifiedinto three subgroups: large-artery atherosclerosis (LAA), small-artery occlusion (SAO), and cardioembolism (CE). Onset time, laboratory test results, and clinical symptoms were compared among the three groups to investigate the rhythmic variations across different TOAST subtypes.

**Results:**

A total of 722 AIS patients were enrolled. For diurnal distribution, LAA patients had higher incidence in early morning, while CE patients showed clustered onset at night. In seasonal distribution, LAA and CE had higher incidence in Quarter 1, whereas SAO patients had higher incidence in Quarter 3. Notable differences existed in onset patterns between weekdays and holidays: significant morning onset peak on weekdays, but irregular onset rhythm on holidays.

**Conclusion:**

This study identified the distribution patterns of different TOAST subtypes of cerebral infarction across circadian rhythm, seasonal variation, and weekdays/holidays differences, thereby providing a valuable reference for medical institutions to develop subtype-specific, rhythm-aligned, season-adapted precise prevention strategies.

## Introduction

Acute ischemic stroke (AIS) is an acute cerebrovascular disease caused by intracranial artery stenosis or occlusion, which can lead to neurological impairments across motor, language, and sensory domains [1]. It is characterized by high incidence, disability, and mortality rates, imposing a significant health and socioeconomic burden worldwide [2].

The Trial of Org 10172 in Acute Stroke Treatment (TOAST) classification is a widely used clinical system for categorizing ischemic stroke, dividing cerebral infarction into five subtypes: large-artery atherosclerosis (LAA), cardioembolism (CE), small-artery occlusion (SAO), stroke of other determined etiology (SOE), and stroke of undetermined etiology (SUE) [3]. Each subtype differs in etiology, risk factors, lesion distribution, and clinical severity, and treatment strategies must be tailored accordingly. Accurate identification of the underlying subtype is therefore essential for optimizing patient management and predicting prognosis [4].

The onset patterns of ischemic stroke are closely linked to alterations in circadian rhythmicity and seasonal trends [5–7]. In addition, differences in onset patterns between weekdays and holidays have rarely been investigated. Exploring circadian rhythmicity, seasonal trends, and the differential impact of weekdays versus weekends on the onset patterns of each TOAST subtype can enable more efficient formulation of pre-hospital care pathways and optimized resource allocation for AIS management.

This study recruited a consecutive cohort of patients with acute ischemic stroke (AIS) presenting to the emergency stroke center, aiming to characterize circadian variations in stroke onset according to TOAST classification.

## Materials and methods

This was a single-center retrospective cohort study. The study was performed at the Emergency Stroke Center of the 960th Hospital of the Joint Logistic Support Force, Jinan, China. The reporting of this observational study followed the STROBE guideline for cohort studies. The study was approved by the Medical Ethics Committee of the 960th Hospital (Approval No. 2025−169).

The collection of pertinent data for comparative studies was initiated on 1/10/2025. The inception cohort was defined a priori as all consecutive patients with AIS admitted to the Emergency Stroke Center of the 960th Hospital of the United Service Support Force from between 1/4/2019 and 30/12/2024

Inclusion criteria: (1) Meet the diagnostic criteria of “Chinese AIS diagnosis and treatment guidelines 2018” [8]. (2) Aged ≥ 18 years old. (3)Onset time < 24h.

Exclusion criteria:(1) Combined with liver and kidney dysfunction. (2) Combined with severe infection. (3) Cannot describe the onset time. (4) Incomplete clinical data.

Relevant baseline patient information was collected, including gender, age, blood pressure, smoking history, drinking history, onset time, admission time, history of underlying diseases (hypertension, diabetes, coronary heart disease), National Institutes of Health Stroke Scale (NIHSS) score, and laboratory results.

According to the classification criteria of TOAST subtypes, the selected patients were classified by TOAST through the relevant medical history and examination results, and divided into five subgroups: LAA group, SAO group, CE group, SOE group, and SUE group. TOAST classification was assessed by two or more attending physicians in the Department of Neurology.

Onset time was defined as the clock time at which focal neurological symptoms were first noticed by the patient or a witness. For wake-up strokes, the last-known-well time was used only when it was clearly documented and within 24 h; otherwise, onset time was considered uncertain and the patient was excluded. Circadian rhythm was predefined as six 4-hour intervals: 00:00–04:00, 04:00–08:00, 08:00–12:00, 12:00–16:00, 16:00–20:00, and 20:00–24:00. Seasonal rhythm was predefined by calendar quarter: Quarter 1 (January-March), Quarter 2 (April-June), Quarter 3 (July-September), and Quarter 4 (October-December). Weekdays were Monday through Friday, excluding statutory public holidays; weekend/holiday included Saturday, Sunday, and statutory public holidays according to the official Chinese calendar for each study year.

### Statistical analysis

Continuous variables were presented as medians (quartiles) and categorical variables as counts and percentages per category. Data were analyzed using SPSS 23.0 software and considered statistically significant at *P* < 0.05. Among them, Chi-square test was used to analyze differences in qualitative indicators between groups; Mann-Whitney U was used to analyze differences in quantitative indicators between groups. Chi-square test was used to analyze the distribution association between TOAST subtypes and time period and season of onset. When an association was statistically significant, standardized residuals were calculated to identify categories contributing most to the association; residuals with an absolute value ≥1.96 were defined as the criterion for abnormal frequency distribution (increased or decreased incidence) in corresponding cell. Analyses were exploratory, and no multivariable adjustment or multiplicity correction was performed. Statistical significance was defined as two-sided P < 0.05. Analyses were performed with SPSS 23.0 and R 4.2.3 using the packages “haprese2” and “ggplot2.”

## Results

### The clinical characteristics of included patients

**Fig 1:**
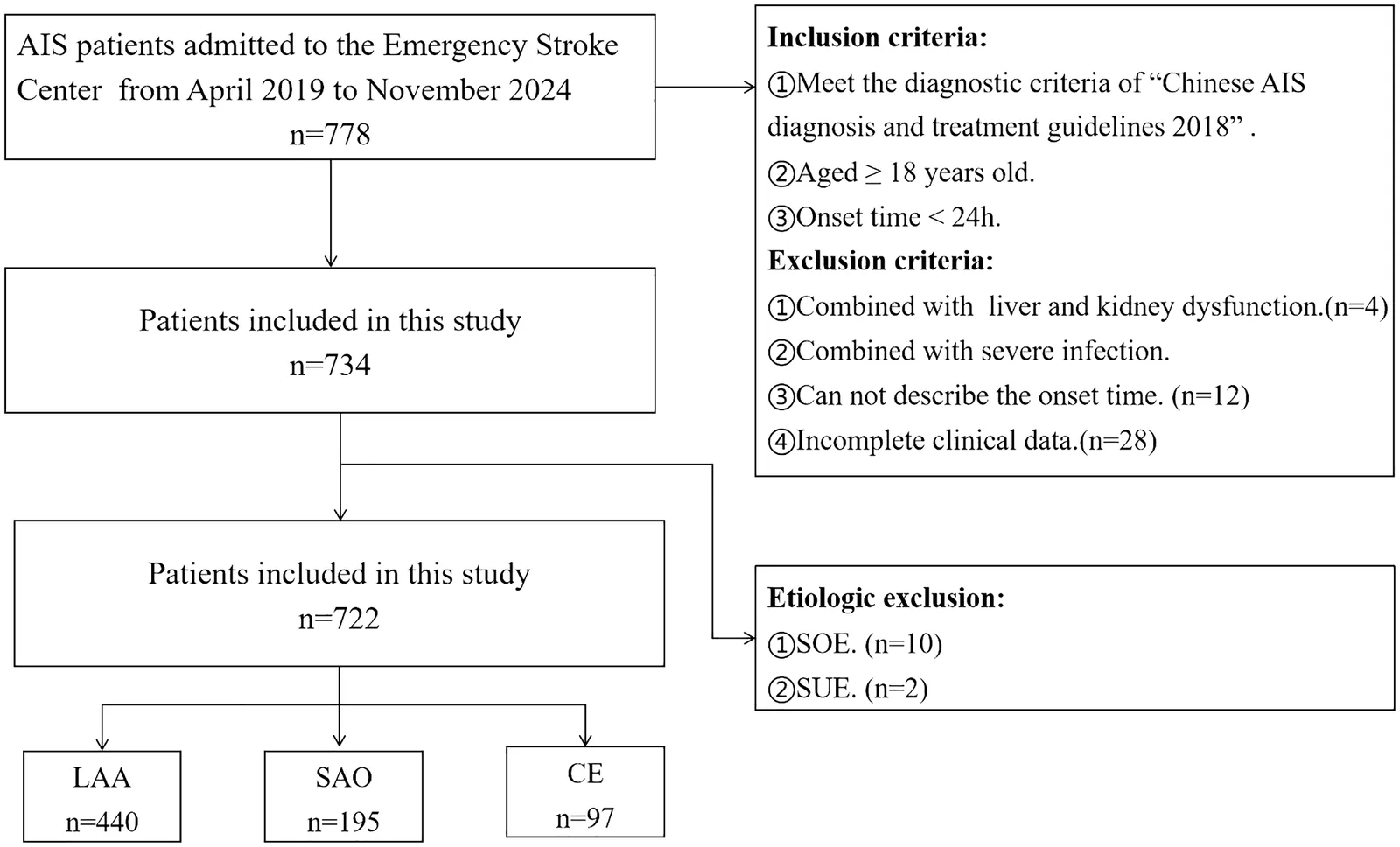
Inclusion and exclusion criteria for patients. Abbreviations: LAA large-artery atherosclerosis, SAO small-artery occlusion, CE cardioembolism.

A total of 778 patients with AIS admitted to the Emergency Stroke Center from April 2019 to November 2024 were initially enrolled. After excluding 44 patients based on the predefined criteria, 734 patients remained for analysis. The final distribution according to the TOAST classification was: 440 with LAA, 195 with SAO, 87 with CE, 10 with SOE, and 2 with SUE**.** Because SOE and SUE accounted for only 12 cases and were too sparse for reliable rhythm-category comparisons, the primary analyses included the three major TOAST subtypes only (LAA, SAO, and CE; n = 722) (Fig 1), and the baseline characteristics of the three groups are shown in Table 1. Compared with LAA and SAO groups, patients with CE were oldest (71.00 [65.00,78.00], *P* < 0.01), had the highest proportion of women (χ^2^ = 31.20, *P* < 0.01), and the highest incidence of coronary heart disease (χ^2^ = 55.59, *P* < 0.01). The CE group had the highest NIHSS scores both at initial diagnosis (12.00 [8.50–17.00]) and after venous thrombolytic therapy compared to the other subtypes (5.00 [2.00–10.00], *P* < 0.01).The results of laboratory indicators indicated that the CE group had the lowest platelet (PLT) count (176.00 [147.25, 221.50]×109/L, *P* < 0.01) and the highest D – dimer level (1.42 [0.90, 2.21], *P* < 0.01). For LAA patients, they had the lowest high density lipoprotein cholesterol (HDL-C) level (1.04 [0.90,1.22], *P* = 0.03) and the highest glycated hemoglobin (HbA1C) level (6.50[5.70,8.20], *P* < 0.01).

**Table 1:** Clinical characteristics of 722 AIS patients.

Variables	Total (n = 722)	LAA (n = 440)	SAO (n = 195)	CE (n = 87)	Statistic	*P*
**Age, M (Q^_1_^, Q_3_)**	65.00 (56.00, 73.00)	65.00 (57.00,73.00)	60.00 (54.00,69.00)	71.00 (65.00,78.00)	χ²=42.26#	**<.01** ^**^
**Sex, No. (%)**					χ²=31.20	**<.01** ^**^
**Male**	491 (68.10)	318 (72.27)	137 (70.26)	36 (41.86)		
**Female**	230 (31.90)	122 (27.73)	58 (29.74)	50 (58.14)		
**Comorbidity, No. (%)**						
**Hypertension**	446 (61.77)	276 (62.73)	113 (57.95)	57 (65.52)	χ²=1.89	0.39
**Diabetes**	195 (27.01)	127 (28.86)	48 (24.62)	20 (22.99)	χ²=2.05	0.36
**Coronary heart disease**	122 (16.90)	61 (13.86)	22 (11.28)	39 (44.83)	χ²=55.59	**<.01** ^**^
**Tobacco Use, No. (%)**	376 (52.08)	251 (57.05)	94 (48.21)	31 (35.63)	χ²=14.95	**<.01** ^**^
**Alcohol Use, No. (%)**	354 (49.03)	234 (53.18)	89 (45.64)	31 (35.63)	χ²=10.18	**<.001** ^***^
**Onset to ED arrival time** **(min), M (Q_1_, Q_3_)**	120.00 (60.00, 205.00)	128.00 (66.50,240.00)	112.50 (58.50,195.00)	90.00 (53.75,157.25)	χ²=13.10#	**<.01** ^**^
**Door to needle time(min), M (Q_1_, Q_3_)**	48.00 (35.00, 74.00)	49.00 (35.00,75.00)	45.00 (34.00,67.00)	63.50 (37.00,91.50)	χ²=4.36#	0.11
**Systolic BP (mmHg), M (Q_1_, Q_3_)**	144.00 (130.00, 158.00)	142.00 (130.00,159.00)	149.00 (135.00,158.75)	139.50 (123.00,153.00)	χ²=10.22#	**<.01**
**Diastolic BP (mmHg), M (Q_1_, Q_3_)**	85.00 (76.00, 93.00)	85.00 (76.00,93.00)	87.50 (80.00,95.00)	80.50 (68.25,91.00)	χ²=13.57#	**<.01** ^**^
**NIHSS-Early, M (Q_1_, Q_3_)**	5.00 (2.00, 11.00)	6.00 (3.00,12.00)	2.00 (1.00,5.00)	12.00 (8.50,17.00)	χ²=129.33#	**<.01** ^**^
**NIHSS-Post, M (Q_1_, Q_3_)**	2.00 (1.00, 5.00)	3.00 (1.00,6.00)	1.00 (0.00,2.00)	5.00 (2.00,10.00)	χ²=65.32	**<.01** ^**^
**Day type, No, (%)**					χ²=0.36	0.83
**Weekday**	504 (69.81)	309 (70.23)	133 (68.21)	62 (71.26)		
**Weekend/holiday**	218 (30.19)	131 (29.77)	62 (31.79)	25 (28.74)		
**Quarter,No, (%)**					χ²=16.82	**<.01** ^**^
**Quarter 1**	187 (25.90)	120 (27.27)	39 (20.00)	28 (32.18)		
**Quarter 2**	190 (26.32)	107 (24.32)	54 (27.69)	29 (33.33)		
**Quarter 3**	198 (27.42)	119 (27.05)	67 (34.36)	12 (13.79)		
**Quarter 4**	147 (20.36)	94 (21.36)	35 (17.95)	18 (20.69)		
**Onset time period, No. (%)**					χ²=20.25	**0.03** ^*^
**0:00-4:00**	54 (7.48)	40 (9.09)	13 (6.67)	1 (1.15)		
**4:00-8:00**	134 (18.56)	88 (20.00)	28 (14.36)	18 (20.69)		
**8:00-12:00**	164 (22.71)	99 (22.50)	51 (26.15)	14 (16.09)		
**12:00-16:00**	143 (19.81)	85 (19.32)	37 (18.97)	21 (24.14)		
**16:00-20:00**	141 (19.53)	84 (19.09)	42 (21.54)	15 (17.24)		
**20:00-24:00**	86 (11.91)	44 (10.00)	24 (12.31)	18 (20.69)		
**Laboratory testing results**						
**HbA1c(%), M (Q_1_, Q_3_)**	6.20 (5.60, 7.80)	6.50 (5.70,8.20)	6.00 (5.60,7.60)	5.90 (5.65,6.50)	χ²=10.95	**<.01** ^**^
**GLU(mmol/L), M (Q_1_, Q_3_)**	5.93 (5.07, 7.92)	6.12 (5.24,8.24)	5.51 (4.89,7.00)	6.39 (5.02,7.97)	χ²=11.80	**<.01** ^**^
**PLT(10** ^**9**^ **/L), M (Q_1_, Q_3_)**	205.50 (168.00, 243.25)	209.00 (174.00,250.00)	207.50 (173.25,241.25)	176.00 (147.25,221.50)	χ²=12.82	**<.01** ^**^
**APTT(s)s, M (Q_1_, Q_3_)**	28.80 (26.80, 31.30)	28.45 (26.70,31.10)	29.70 (26.98,31.52)	29.70 (27.30,32.78)	χ²=5.62	0.06
**D-dimer (mg/L), M (Q_1_, Q_3_)**	1.12 (0.53, 2.26)	1.10 (0.53,2.33)	0.89 (0.33,1.62)	1.42 (0.90,2.21)	χ²=9.54	**<.01** ^**^
**PT/INR, M (Q_1_, Q_3_)**	11.13 (10.97, 11.52)	11.12 (10.96,11.52)	11.43 (11.00,11.54)	11.12 (11.00,11.52)	χ²=1.64	0.44
**TC(mmol/L), M (Q_1_, Q_3_)**	4.59 (3.83, 5.32)	4.60 (3.81,5.33)	4.61 (3.94,5.36)	4.38 (3.66,5.05)	χ²=2.42	0.30
**TG(mmol/L), M (Q_1_, Q_3_)**	1.13 (0.83, 1.66)	1.15 (0.83,1.66)	1.22 (0.91,1.80)	0.94 (0.59,1.24)	χ²=17.06	**<.01** ^**^
**HDL-C(mmol/L), M (Q_1_, Q_3_)**	1.07 (0.91, 1.23)	1.04 (0.90,1.22)	1.08 (0.91,1.27)	1.14 (0.98,1.30)	χ²=6.95	**0.03** ^*^
**LDL-C(mmol/L), M (Q_1_, Q_3_)**	2.80 (2.18, 3.55)	2.83 (2.19,3.60)	2.78 (2.20,3.56)	2.66 (2.07,3.25)	χ²=3.87	0.14
**HCY(μmol/L), M (Q_1_, Q_3_)**	11.80 (9.50, 15.80)	11.75 (9.60,15.60)	11.50 (9.70,15.80)	12.10 (8.90,16.30)	χ²=0.14	0.93
**ADA(U/L), M (Q_1_, Q_3_)**	11.30 (9.25, 14.00)	11.60 (9.40,14.50)	11.00 (9.10,13.00)	11.90 (10.00,15.00)	χ²=7.20	**0.03** ^*^
**TyG, M (Q_1_, Q_3_)**	2.26 (0.00, 4.48)	2.24 (0.00,4.53)	2.54 (0.00,4.61)	1.83 (0.00,3.49)	χ²=2.81	0.25

Abbreviations:AIS Acute ischemic stroke, LAA large-artery atherosclerosis, SAO small-artery occlusion, CE cardioembolic, ED Emergency Department, BP Blood Pressure, NIHSS National Institute of Health stroke scale, HbA1c Glycated hemoglobin, GLU Glucose, PLT Platelet, APTT Activated Partial Thromboplastin Time, PT Prothrombin Time, INR International Normalized Ratio,TC Total Cholesterol, TG Triglyceride, HDL-C High density lipoprotein cholesterol, LDL-C Low-Density Lipoprotein Cholesterol, HCY Homocysteine, AD Adenosine Deaminase TyG triglyceride-glucose index

M:Median, Q_1_ 1st Quartile, Q_3_ 3st Quartile, χ²: Chi-square test,*:*P* < 0.5 **:*P* < 0.01 ***:*P* < 0.001

### Circadian variation of the three subtypes

**Fig 2:**
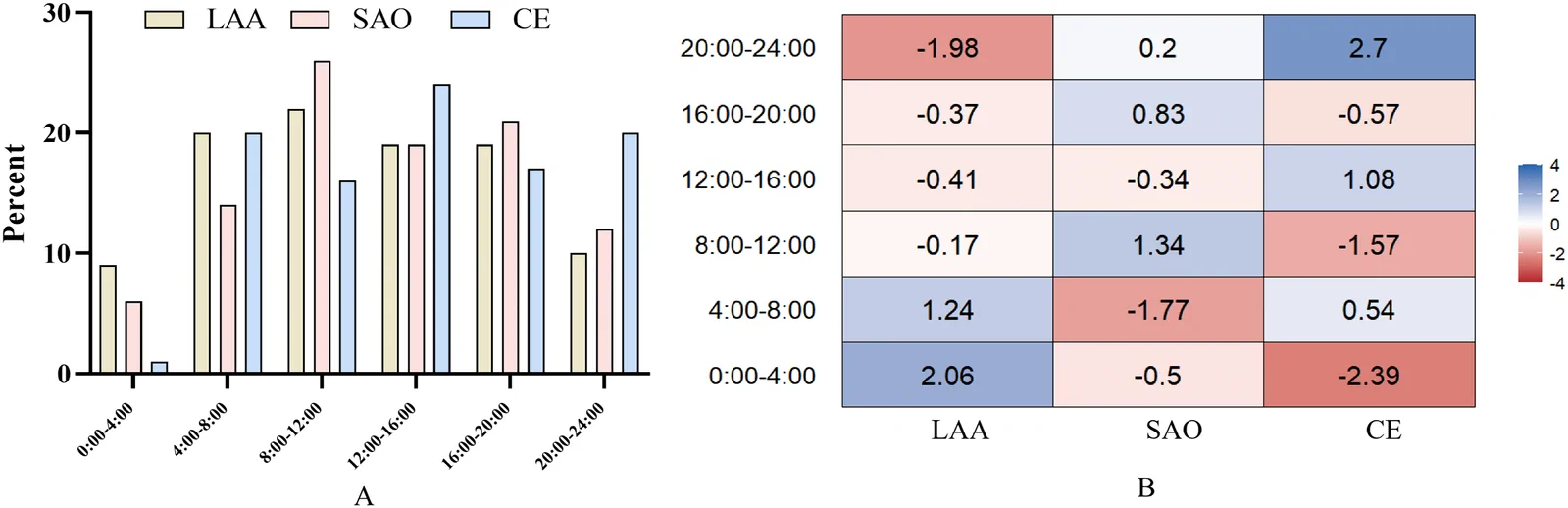
The incidence proportion in each time period according to the three subtypes. (A).Each bar chart represents the onset proportion of each subtype within the corresponding time period. (B). Matrix showing the number of patients of each T subtype in their respective time slot and the theoretically expected number of cases, in relation to the observed cases of other subtypes. Blue is associated with a positive correlation, and red is associated with a negative distribution. Abbreviations: LAA large-artery atherosclerosis, SAO small-artery occlusion, CE cardioembolism.

The chi-square test was used to analyze the distribution of the three groups across six time periods. A significant association was found between the time of onset and stroke subtype (χ^2^ = 20.25, *P* = 0.03) (Table 1). Fig 2A illustrates that AIS patients had a higher incidence between 4:00 and 20:00 throughout the day, with the highest peak between 8:00 and 12:00 hours. There exist differences in the distribution of the three groups across different time periods. Standardized residual analysis was employed to further uncover abnormal distribution patterns during specific time periods (Fig 2B). During the 0:00–4:00 period, the LAA group was significantly more prevalent (residual = 2.06), whereas the CE group was significantly less prevalent (residual = −2.39). From 20:00–24:00, the LAA group exhibited a significantly lower incidence (residual = −1.98), while the CE group had a significantly higher incidence (residual = 2.7). No significant differences in incidence rates were observed among the three subtypes during the other time periods (4:00–8:00, 8:00–12:00, 12:00–16:00, 16:00–20:00).

### Seasonal variation of the three subtypes

**Fig 3:**
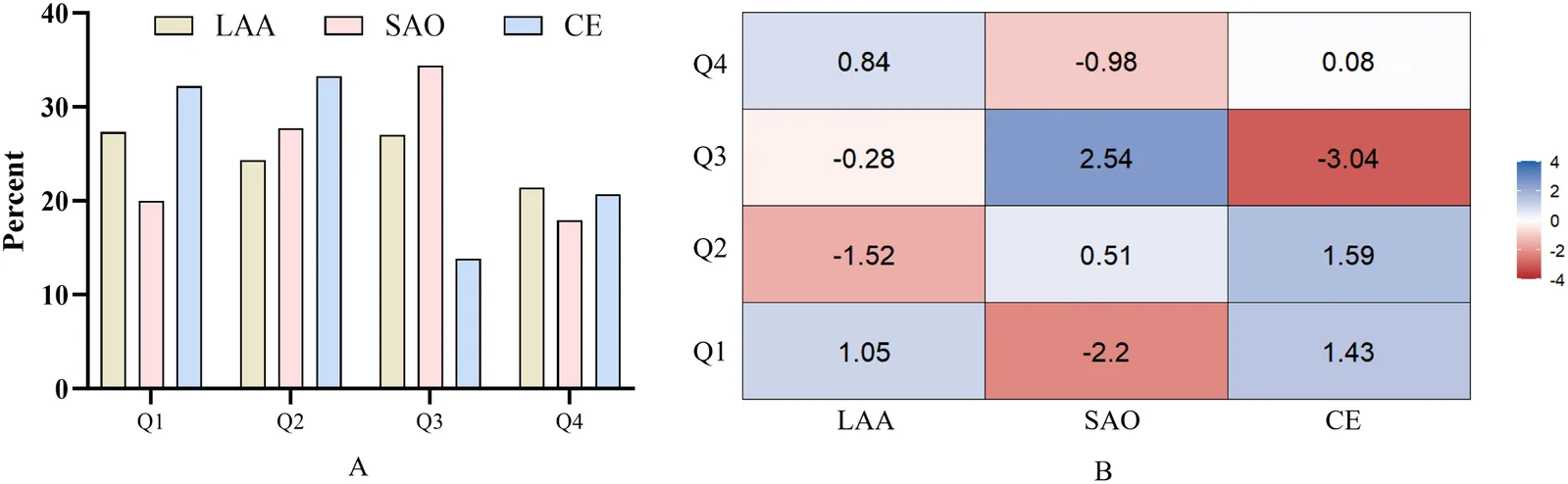
The incidence proportion in each quarter according to the TOAST subtype. (A). Each bar chart represents the incidence proportion of each subtype in the corresponding quarter. (B). Matrix showing the number of patients of each subtype in their respective quarter and the theoretically expected number of cases, in relation to the observed cases of other subtypes. Blue is associated with a positive correlation, and red is associated with a negative distribution. Abbreviations: LAA large-artery atherosclerosis, SAO small-artery occlusion, CE cardioembolism.

There was a significant association between season of onset and stroke classification (χ2 = 16.82, P < 0.01). Post-hoc analysis of standardized residuals identified specific seasonal patterns: the prevalence of SAO group was significantly lower in Quarter 1 (residual = −2.2) but higher in Quarter 3 (residual = 2.54). Conversely, the CE group was significantly less prevalent in Quarter 3 (residual = −3.04). No significant differences were found in the incidence of the three subtypes between Quarter 2 and Quarter 4. (Fig 3)

### Weekday vs holidays pattern variation of the three subtypes

**Fig 4:**
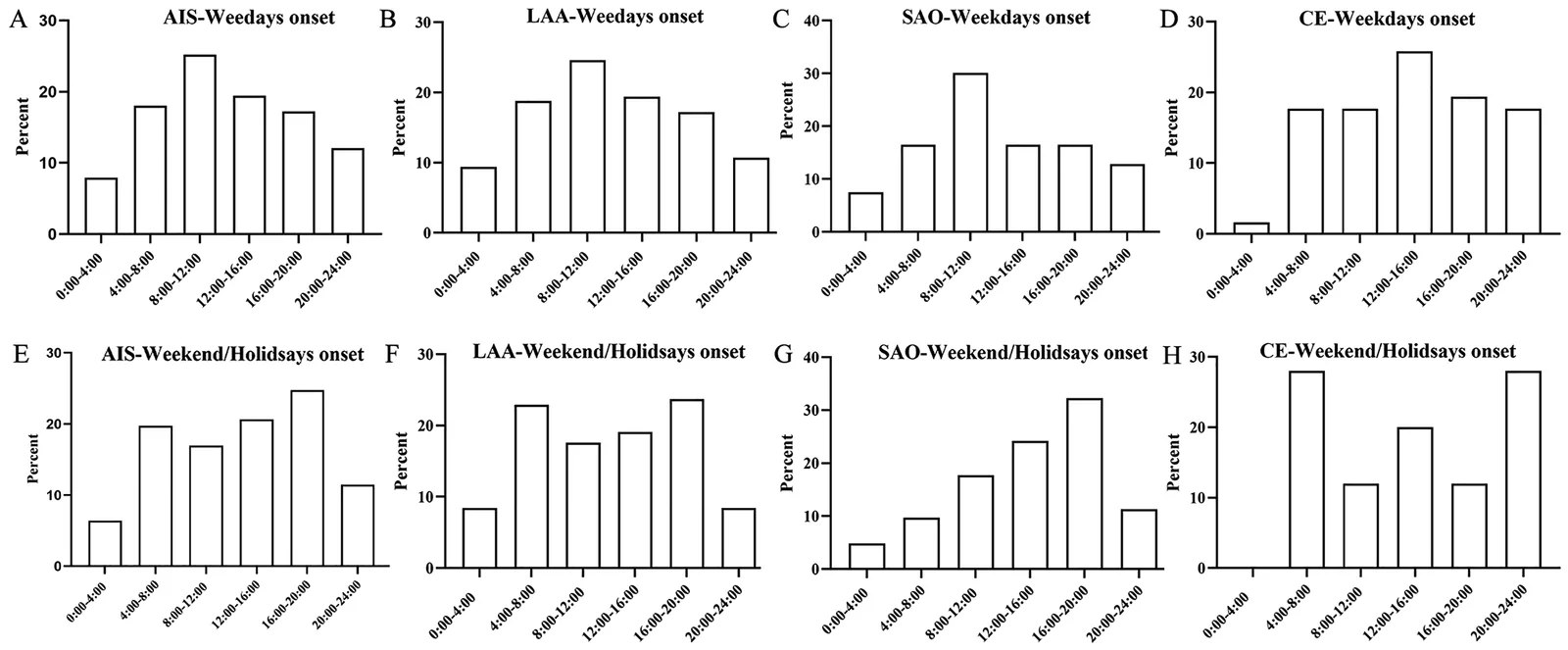
Weekdays vs weekends/holidays, proportion with time of stroke onset in each each four-hour interval of day-night cycle. (A) – (D). For patients with different subtypes, bar chart displays conveying magnitudes of onset in each four-hour for weekdays. (E) – (H). For patients with different subtypes, bar chart displays conveying magnitudes of onset in each four-hour for weekends/holidays. Abbreviations: LAA large-artery atherosclerosis, SAO small-artery occlusion, CE cardioembolism.

For patients with AIS, the incidence of AIS presents a single peak during 8:00–12:00 on weekdays, and a wide plateau during 4:00–20:00 on holidays. There are differences in the distribution patterns of the three subtypes. For LAA groups, the onset of disease on weekdays showed a single peak pattern from 8:00–12:00, and the onset of disease on holidays showed a double peak pattern, which was at 4:00–8:00 and 16:00–20:00, respectively. For SAO groups, the incidence of both weekdays and holidays showed a single peak pattern, with weekdays in the earlier period of 8:00–12:00 and holidays in the period of 16:00–20:00. For the CE groups, the onset of disease on weekdays showed a single peak pattern from 12:00–16:00, and the onset of disease on holidays showed a double peak pattern, which was at 4:00–8:00 and 20:00–24:00, respectively (Fig 4).

## Discussion

The main findings of this retrospective cohort study were as follows. First, onset time distribution differed across TOAST subtypes: LAA was overrepresented in the 00:00–04:00 interval, whereas CE was overrepresented in the 20:00–24:00 interval. Second, seasonal distribution differed by subtype, with SAO overrepresented in Q3 and CE underrepresented in Q3. Third, AIS onset showed a clear weekday morning peak, whereas weekend/holiday onset was broader and less regular. Together, these results suggest that different etiological subtypes of AIS may be influenced by distinct circadian, seasonal, and behavioral contexts.

The baseline characteristics of the three subtypes were consistent with their known clinical profiles and support the biological plausibility of the rhythm findings. CE is more common in elderly women, often accompanied by coronary heart disease and severe neurological deficits, which are directly related to its etiological characteristics. CE is mostly caused by diseases that produce cardiac thrombi, such as atrial fibrillation and valvar heart disease, which are more common in the elderly and women. Moreover, embolic lesions often involve large vessels, leading to more extensive cerebral ischemia and more severe neurological damage [9]. The decreased PLT and elevated D-dimer levels in CE patients suggest a more significant activation of the coagulation-fibrinolysis system, which may be related to the enhanced fibrinolytic response following thrombosis [10,11]. Additionally, the lower HDL-C and higher HbA1c levels in LAA patients further confirm the close association between atherosclerosis and metabolic abnormalities, such as diabetes and dyslipidemia. The vascular lesions in these patients are often caused by the progression of atherosclerosis due to long-term lipid metabolism disorders and poor blood glucose control [12–14]. SAO patients are relatively younger, more likely to be male, and have a higher proportion of hypertension. This may be related to arteriolosclerosis or the formation of microaneurysms, which often occur in patients with poorly controlled long-term hypertension, leading to occlusion of perforating arteries and lacunar infarction [15,16]. Consequently, these subtype-specific baseline characteristics support a more individualized approach to AIS management. CE patients may benefit from prompt cardiac source screening and anticoagulation evaluation, LAA patients from aggressive metabolic control and vascular assessment, and SAO patients from sustained hypertension management and small-vessel protection.

In this study, we found a significant association between TOAST subtypes of ischemic stroke and time period of onset. The LAA group had the highest incidence compared with the SAO group and the CE group during the 0:00–4:00 time period, which may be closely related to the changes of human physiological rhythm. Increased sympathetic tone in the morning hours, early initiation of the “morning surge phenomenon” in diurnal fluctuations in blood pressure, increased blood viscosity, and increased platelet aggregation may promote instability and thrombosis of atherosclerotic plaques in large arteries, which is consistent with previous mechanistic studies on the high incidence of atherosclerotic vascular events in the early morning hours [17–20]. The significant accumulation of CE between 20:00 and 24:00 may be related to the relatively increased vagal tone during this period, resulting in altered heart rate variability, reduced atrial systolic function, and increased risk of mural thrombus detachment in patients with atrial fibrillation [21]. Accordingly, characterizing the circadian differences in the incidence patterns of AIS and its TOAST subtypes may help stroke centers anticipate subtype-related prevention, diagnostic, and therapeutic needs. For LAA-prone patients, stricter early-morning control of blood pressure, platelet activation, lipid levels, and atherosclerotic risk factors may reduce plaque instability and thrombosis.For CE-prone patients, evening rhythm monitoring, timely identification of atrial fibrillation, and appropriate anticoagulation assessment should be strengthened.

A significant relationship was identified between TOAST classification and the seasonal onset of AIS in our cohort., revealing differences in the sensitivity of different etiological subtypes to seasonal environments. In Quarter 1, LAA and CE are relatively high and may be associated with increased cold-induced vasoconstriction, hemodynamic stress synergizing with inflammatory responses, lipid metabolism disorders, and jointly promoting arterial thrombosis and increased frequency of atrial fibrillation episodes [22–24]. Decreased physical activity or increased respiratory infections, which exacerbate inflammation and thrombosis, may contribute to the development of disease during cold seasons [25,26]. In Quarter 3, the high incidence of SAO is mainly attributed to the high evaporation of body fluids caused by the high temperature environment in summer, which in turn causes increased blood viscosity and exacerbates the degeneration of cerebral arterioles [27,28]. At the same time, increasing summer temperatures reduce the incidence of CE by reducing afterload and reducing left atrial appendage thrombosis [29]. In addition, this study also found no significant difference in the incidence of the three subtypes between Quarter 2 and Quarter 4, which may be related to the moderate temperature in spring and autumn and the relative balance of vasoconstriction and relaxation status. Taken together recognizing the seasonal differences in the incidence patterns of AIS and its TOAST subtypes may help stroke centers anticipate subtype-specific diagnostic and therapeutic needs, such as enhanced vascular imaging and antithrombotic management during winter LAA-enriched periods, strengthened cardiac rhythm monitoring and anticoagulation evaluation during CE-enriched periods, and improved hydration guidance and small-vessel risk management during summer SAO-enriched periods.

The incidence patterns of AIS on weekdays and holidays are different, which may be attributed to the changes in patients’ sleep-wake rhythms and biological states. On weekdays, people usually follow a more regular schedule, and the onset of AIS is concentrated in the morning (8:00–12:00), which is consistent with the classic physiological rhythms such as the morning blood pressure surge, sympathetic nerve activation, and hypercoagulable state of blood [30,31]. On holidays, the schedule may be more flexible, with more variable sleep and activity times. This change in the daily routine may lead to the disruption of the biological clock, thereby affecting physiological functions and increasing the risk of AIS. At the same time, the work pressure and tense atmosphere on weekdays may also have an impact on an individual’s psychological and physiological states, further increasing the risk of AIS. The stable single-peak pattern of SAO (morning peak on weekdays / evening peak on holidays) suggests that it is more dependent on the basic blood pressure rhythm, and the evening peak on holidays may be related to the delayed hypertension response caused by increased nighttime activities [32]. The midday peak (12:00–16:00) of CE on weekdays coincides with the period of increased sympathetic tension in patients with atrial fibrillation, and the double-peak pattern on holidays may be related to the detachment of emboli due to changes in sleep posture and increased nighttime activities on weekends [33]. Therefore, understanding the differences in the incidence patterns of AIS and its different subtypes on weekdays and holidays can help help stroke centers anticipate subtype-related diagnostic needs, such as vascular imaging capacity during early-morning LAA-enriched periods and rapid cardiac evaluation pathways during CE-enriched periods.

These findings suggest that the pathogenesis of different ischemic stroke subtypes may be influenced by diverse circadian, seasonal, and behavioral factors, highlighting the potential for chronotherapeutic strategies and subtype-specific prevention approaches. Neurologists can remind high-risk people to avoid rushing to travel during weekdays, reduce blood pressure elevations induced by emotional stress, maintain work and rest times similar to weekdays on holidays, avoid staying up late or excessive sleep, and avoid overeating on their diet, especially avoiding excessive alcohol consumption. Strengthen team response and device activation to respond to LAA subtypes in the early morning (00:00–04:00); strengthen high-risk patient monitoring and open CE subtype fast track at night (20:00–24:00); focus on optimizing targeted diagnosis and treatment of specific subtypes in Quarter 1 and Quarter 3.

This study has the following limitations: 1. This study is a single-center study with a relatively small sample size. In future studies, multicenter studies may be conducted to expand the sample size and thereby validate the conclusions of this study. 2. Conclusions on seasonal distribution may not apply to other regions, particularly southern cities with large differences in climate and environmental conditions, as this study is confined to northern cities and has certain geographical limitations. To gain a comprehensive understanding of the impact of seasonal distribution, subsequent studies should cover a wider geographic area to enhance generalizability and accuracy of conclusions.

## Conclusion

In this single-center retrospective cohort, onset rhythms differed among LAA, SAO, and CE subtypes of AIS. LAA peaked in the early morning and CE at night; LAA and CE were more prevalent in the first quarter, while SAO predominated in the third quarter. Weekday onset presented a morning peak, and holiday onset was irregular. These exploratory findings support the need for multicenter validation and may inform subtype-specific prevention counseling and rhythm-aligned stroke-center preparedness.

## Supporting information

S1 Data Raw data. (XLSX)
